# Portable rotating grating stimulation for anisometropic amblyopia with 6 months training

**DOI:** 10.1038/s41598-021-90936-7

**Published:** 2021-06-01

**Authors:** Wen-Hsiu Yeh, Li-Ju Lai, Da-Wei Chang, Wei-Sin Lin, Guan-Ming Lin, Fu-Zen Shaw

**Affiliations:** 1 Institute of Basic Medical Sciences, National Cheng Kung University, Tainan, 701 Taiwan; 2 Department of Ophthalmology Chang Gang Memorial Hospital Chia-Yi Branch, Chiayi, 613 Taiwan; 3 Department of Computer Science and Information Engineering, National Cheng Kung University, Tainan, 701 Taiwan; 4 Department of Psychology, National Cheng Kung University, No 1 University Road, Tainan, 70101 Taiwan

**Keywords:** Randomized controlled trials, Paediatric research, Eye manifestations, Rehabilitation

## Abstract

Treatment of grating stimulation has been used in amblyopia for decades, but high dropout rate and inconvenience for daily practice occur in previous studies. We developed a home-based portable system with rotating grating stimulation on a tablet. Thirty anisometropic amblyopic children were randomly allocated into the control or Grating group. They drew contour of the picture under patch of a better eye for 6 months. Best-corrected visual acuity (BCVA), grating acuity (GA), and contrast sensitivity (CS) were assessed at the baseline, 1st, 2nd, 3rd, and 6th months of training. All participants completed the 6-month training. Patched eyes of both groups exhibited no difference. Trained eyes of the control group had significantly slight improvement in BCVA and GA. In particular, the Grating group exhibited significantly higher BCVA, GA, and CS compared with those of the control group at the 3rd and 6th months of training. Moreover, percentage of the Grating group with great improvement (BCVA ≥ 0.3 or CS ≥ 0.3) was significantly larger than those of the control group at the 3rd or 6th months of training. The portable grating stimulation system demonstrates its trainability by no dropout and effectiveness by significant improvements in all assessments through a well experimental design.

Trial Registration: ClinicalTrials.gov NCT04213066, registered 30/12/2019, https://clinicaltrials.gov/ct2/show/NCT04213066.

## Introduction

Amblyopia exhibits impaired vision in one or both eyes and affects up to 5% of the population^1^. These patients cannot receive clear images on the retina to develop a proper connection to the visual cortex during the infant period, which often leads to immature development or to grow slowly and abnormally^2^. Amblyopic eye usually exhibits poor visual function measured by visual acuity (VA)^3^, grating acuity (GA)^4^, or contrast sensitivity (CS)^5,6^. A poor visual function is always accompanied by inattentive behavior, which easily leads to fall down frequently or lower learning ability^7^. Moreover, the patient usually develops a learned non-use strategy for amblyopic eye then results in reducing one’s eye contact during personal communication^8^. Thus, an effective treatment is needed for amblyopic people.

Full-time occlusion therapy has been used for decades but an educationally disruptive technique resulting in high dropout rate^9^. Thereafter, a Cambridge (CAM) vision stimulator with part-time occlusion has been proposed to force using amblyopic eye under grating stimuli of various spatial frequencies for 10–60 min a day lasting for 2–6 months^9–12^. Effectiveness of CAM stimulation remains largely controversial for decades^9,13–15^. Several factors account for these varied inconclusive results for the CAM treatment. For instance, previous studies are often absence of a control group^9,16^ or design an inadequate control group^14^. Randomization and better match group are beneficial to explore effectiveness of the CAM stimulation. To determine advantage of the CAM stimulation on amblyopia, a better clinical design is necessary. Previous CAM studies designed with adequate control group express inconclusive advantage for amblyopia^17,18^. Acute or short-term treatment of CAM stimulation has little effect on amblyopia^13,19^. Long-term treatment (> 2 months) seems to be more effective for CAM treatment even their results are inconclusive^20,21^.

Amblyopic children usually lose their interests or drop out frequently because of stereotyped drawing with little change in a general CAM stimulator^16^ or only small or slow visual improvement^17^. The CAM stimulator^9^ or other treatments^22,23^ are commonly set up in a hospital or clinic. Patients and their parents have to visit the hospital several times per week regularly. Recently, numerous studies have proposed grating stimuli incorporated into computer games instead of classic drawings^1,21^. Computer or notebook has largely inconvenience for children due to a bulk size and heavy weight compared with tablet^24^. Most of the video games for amblyopia are absence^24^ or only presence of a single-frequency grating stimulation^21^. These studies present a considerable dropout rate during the training^21,24^. A meta-analysis study has indicated small sample size (n < 10) in previous video game studies with small VA improvement (0.13–0.21 logMAR)^25^. These video game studies evaluate VA of amblyopia exclusively. A systematic evaluation in different aspects of visual functions will be important for effectiveness of CAM stimulator in amblyopia.

To ascertain effectiveness and convenience of CAM device, a home-based system may be a choice to save time and reduce dropout rate. To address these issues, a home-based amblyopic training system on a tablet with a variety of stimuli (including grating patterns and pictures) might save time for attending a hospital and provide flexible training schedule for any place (e.g., journey). Practice for frequency and the orientation of the gratings is crucial to improve visual functions, e.g., CS^26^. A self-realization flow^27^ could be considered in the tablet to increase motivation of amblyopic children and their parents. Importantly, the present study constructed two groups: the control group received drawing eye-hand training and the experimental group (Grating) received drawing eye-hand practice with rotating grating stimuli of various spatial frequencies. We hypothesized that the Grating group showed significant enhancement of visual function compared with the control group through the home-based training apparatus.

## Results

**Figure 1:**
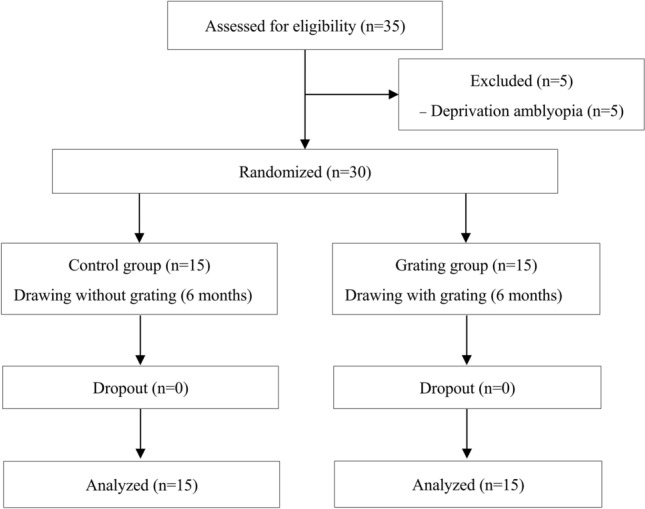
Flow diagram according to the Consolidated Standards of Reporting Trials (CONSORT) statement showing recruitment, randomization, and patient flow in the study.

Thirty participants had completely finished 6 months’ training and evaluation (Fig. 1). Basic characteristics, including age, gender, BCVA, GA and CS, were not significant in patched and trained eyes between the two groups (Table 1). Both groups wore an eye patch over a better-seeing eye during training. The two groups exhibited no significant difference in total training time (control, 45.7 ± 2.2 h; Grating, 42.8 ± 1.5 h; t = 1.1, p = 0.28) and total training sessions (control, 182.6 ± 8.6; Grating, 171.1 ± 6; t = 1.1, p = 0.28). Total training time was the duration of part-time occlusion in both groups.

**Table 1:** Basic characteristics in patched and trained eyes of the control and Grating groups. Data are expressed as mean (standard error of mean). BCVA = best-corrected visual acuity; cpd = cycles per degree; CS = contrast sensitivity; GA = grating acuity. ^a^Student t test. ^b^χ^2^ test.

	Control (n = 15)	Grating (n = 15)	*p* value^a^
Gender (boy:girl)	7:8	5:10	0.71^b^
Age	5.40 (0.32)	5.47 (0.39)	0.90
**Patched eye**			
BCVA	0.14 (0.04)	0.16 (0.04)	0.81
GA	24.93 (1.04)	27.73 (1.17)	0.08
CS (16 cpd)	1.22 (0.21)	1.37 (0.19)	0.59
**Trained eye**			
BCVA	0.28 (0.03)	0.35 (0.06)	0.31
GA	21.67 (0.67)	22.33 (1.32)	0.66
CS (16 cpd)	0.92 (0.09)	0.93 (0.14)	0.97

### Best-corrected visual acuity (BCVA)

**Figure 2:**
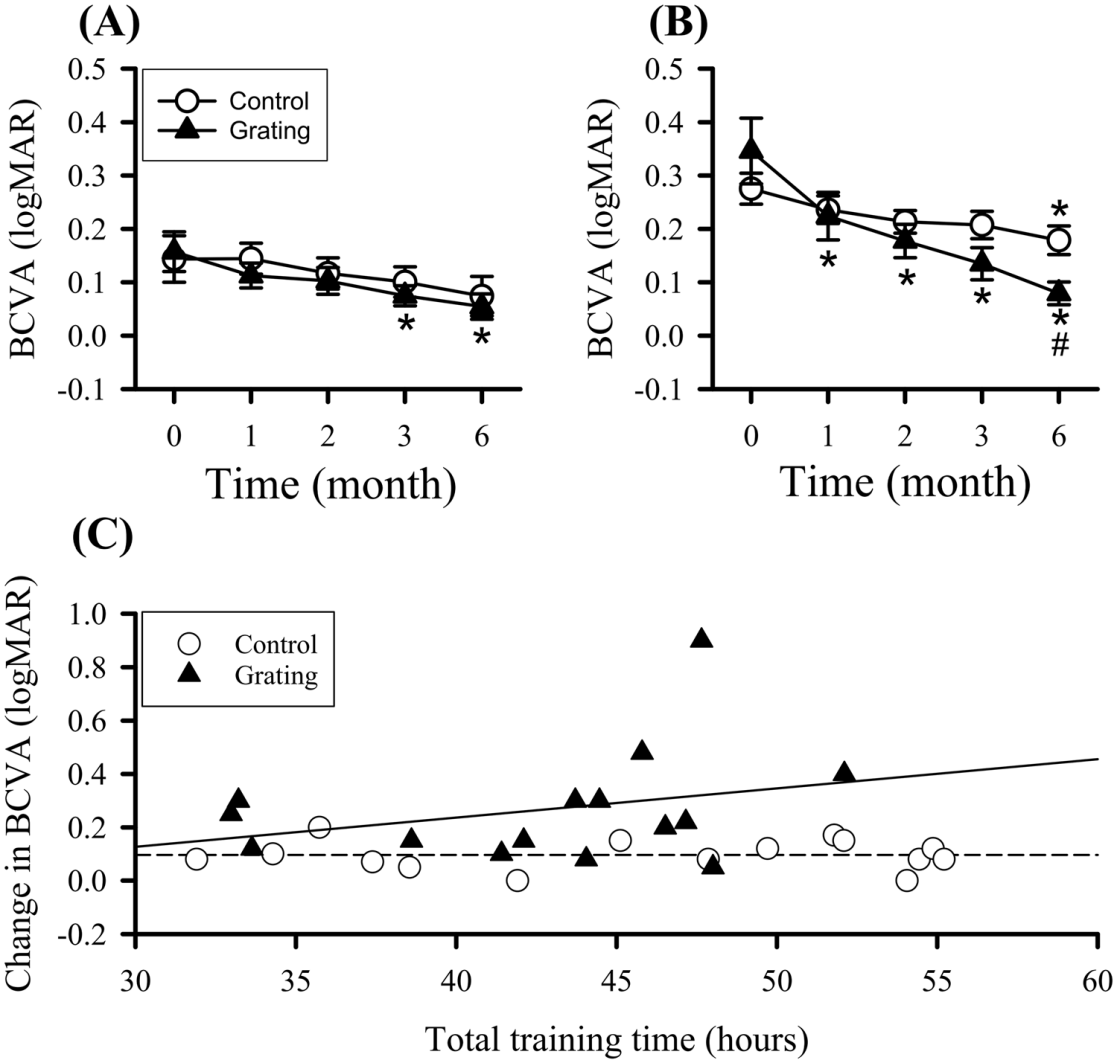
Best-corrected visual acuity (BCVA) at the baseline (0), 1st, 2nd, 3rd, and 6th months after the training. (**A**) BCVAs in patched eyes of the control and Grating groups. (**B**) BCVAs in trained eyes of the control and Grating groups. *p < 0.05 versus baseline, ^*#*^p < 0.05 versus the control group. (**C**) Relationship between BCVA gain from the 6th month to baseline and total training time in trained eye of the two groups. The long dashed (control) and solid (Grating) lines represent the linear regression line fit the data.

Figure 2 shows the BCVA in patched and trained eyes of the two groups throughout the 6-month training. In patched eyes of the two groups (Fig. 2A), BCVA showed significant difference in the factor of time (F(4,109) = 5.93, p < 0.001). BCVA of the control group exhibited no significant difference throughout training. BCVA of the Grating group exhibited significantly better at the 3rd and 6th months compared with its baseline. In trained eyes of the two groups (Fig. 2B), BCVA showed significant difference in the factors of time (F(4,112) = 17.87, p < 0.001) and group × time (F(4,112) = 4.07, p = 0.004). The control group exhibited significantly better BCVA at the 6th month compared with its baseline. BCVA of the Grating group exhibited significantly better at the 1st, 2nd, 3rd and 6th months compared with baseline BCVA. Trained eyes of the Grating group had improved BCVA of − 0.27 ± 0.04 logMAR from baseline to the 6th month. Moreover, trained-eye BCVA of Grating group at the 6th month was significantly better than that in the control group.

Relationship between BCVA gain from the 6th month to baseline and total training time in trained eye of the two groups was analyzed (Fig. 2C). In trained eye of the control group, there was no significant correlation between BCVA gain and total duration (r = 0). In trained eye of the Grating group, there was a slight positive correlation but not attain a significant level (r = 0.3, p = 0.28).

The two groups had no severe deterioration of BCVA throughout the training. The control group exhibited BCVA change (from 0 to − 0.17 logMAR) at the 6th month. All children in the Grating group showed absolute BCVA improvement (from − 0.05 to − 0.48 logMAR) at the 6th month. Participants who gained BCVA improvement of ≤ -0.3 logMAR in trained eyes of the two groups were counted (Table 2). Better BCVA improvement only occurred in ~ 10% of the control group. In contrast, participants with better BCVA improvement exhibited a progressive elevation to 53.3% 6 months after training. Participants with BCVA improvement at the 6th month were significantly different in the two groups (χ^2^(1) = 8.35, p = 0.004). There was no difference in patched eyes of the two groups who gained BCVA improvement (Table S1 in supplementary results).

**Table 2:** Participants have gained better BCVA, GA, and CS of trained eye at the 1st, 2nd, 3rd, and 6th months after the training in the control and Grating groups. ^*#*^p < 0.05 vs. the control group by χ^2^ test.

	Control	Grating
**BCVA (≤ -0.3)**		
1 month	0/15 = 0%	2/15 = 13.3%
2 month	2/15 = 13.3%	3/15 = 20%
3 month	1/15 = 6.7%	4/15 = 26.7%
6 month	0/15 = 0%	8/15 = 53.3%^#^
**GA (≥ 10 cpd)**		
1 month	0/15 = 0%	2/15 = 13.3%
2 month	0/15 = 0%	3/15 = 20%
3 month	2/15 = 13.3%	7/15 = 46.7%
6 month	5/15 = 33.3%	11/15 = 73.3%
**CS (≥ 0.3)**		
1 month	0/15 = 0%	3/15 = 20%
2 month	3/15 = 20%	9/15 = 60%
3 month	3/15 = 20%	10/15 = 66.7%^#^
6 month	3/15 = 20%	12/15 = 80%^#^

### Grating acuity (GA)

**Figure 3:**
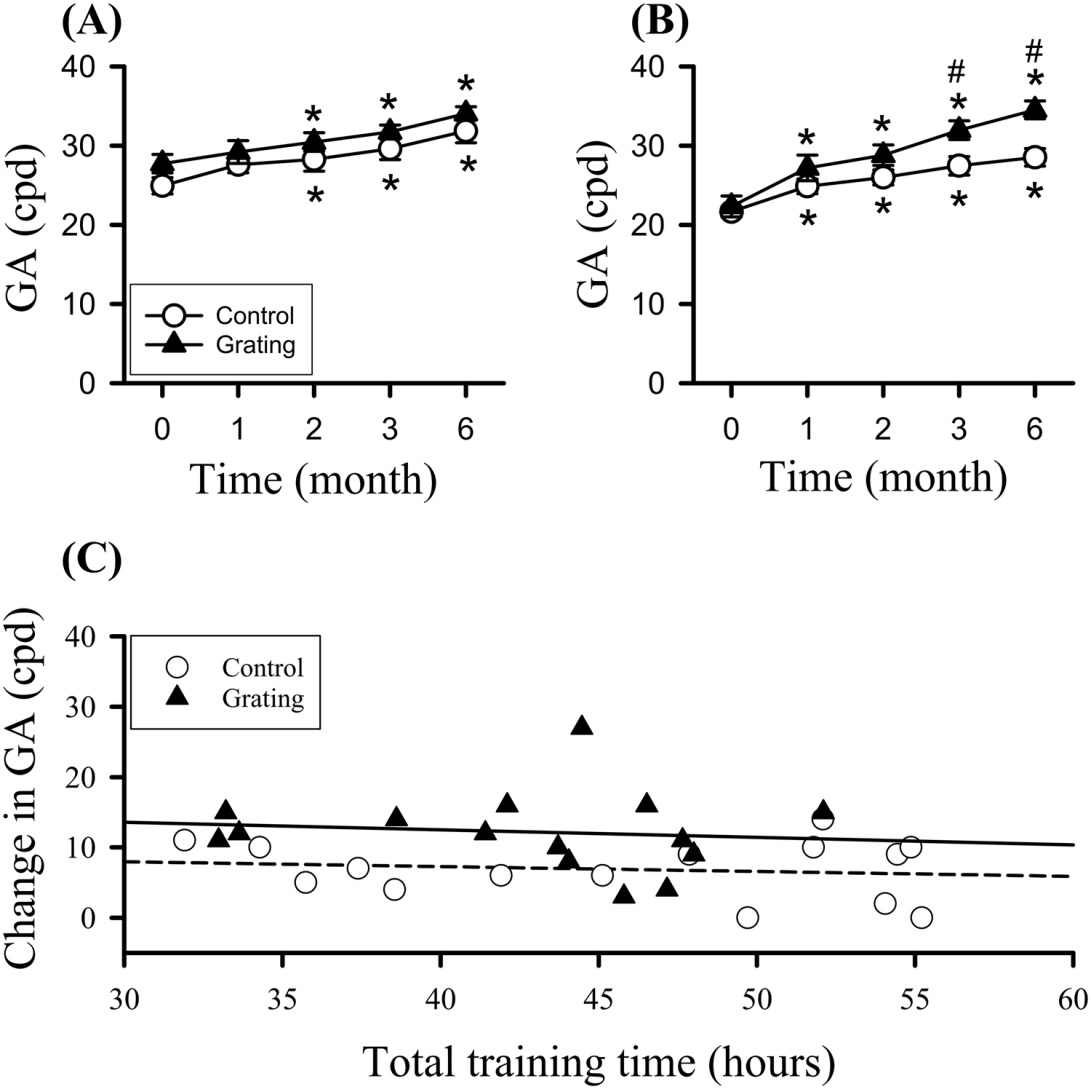
Grating acuity (GA) at the baseline (0), 1st, 2nd, 3rd, and 6th months after the training. (**A**) GAs in patched eyes of the control and Grating groups. (**B**) GAs in trained eyes of the control and Grating groups. *p < 0.05 versus baseline, ^*#*^p < 0.05 versus the control group. (**C**) Relationship between GA gain from the 6th month to baseline and total training time in trained eye of the two groups. The long dashed (control) and solid (Grating) lines represent the linear regression line fit the data.

Figure 3 shows the GA in patched and trained eyes of the two groups throughout the 6-month training. In patched eyes of the two groups (Fig. 3A), GA showed significant difference in the factor of time (F(4,112) = 21.48, p < 0.001). GA of the control group exhibited significantly higher GA at the 2nd, 3rd and 6th months compared with its baseline. GA of the Grating group exhibited significantly higher at the 2nd, 3rd and 6th months compared with baseline GA. In trained eyes of the two groups (Fig. 3B), GA showed significant difference in the factors of time (F(4,112) = 55.55, p < 0.001), group (F(1,28) = 4.29, p = 0.04) and their interaction (F(4,112) = 4.72, p = 0.001). The control group exhibited significantly higher GA at the 1st, 2nd, 3rd and 6th months compared with baseline GA. GA of the Grating group exhibited significantly higher at the 1st, 2nd, 3rd and 6th months compared with its baseline. Trained eye of the Grating group had increased GA of 12.2 ± 1.47 cpd from baseline to the 6th month. Moreover, trained-eye GA of the Grating group at the 3rd and 6th months was significantly higher than that in the control group.

Relationship between GA gain from the 6th month to baseline and total training time in trained eye of the two groups was analyzed (Fig. 3C). In trained eye of the control group, there was no significant correlation between GA gain and total duration (r = -0.1). In trained eye of the Grating group, there was little correlation (r = -0.11).

The two groups had no severe deterioration of GA throughout the training. Participants who gained GA improvement of ≥ 10 cpd in trained eyes of the two groups were counted (Table 2). Increased GA only occurred in at most 33% of the control group. In contrast, participants with better GA improvement exhibited a progressive elevation to 73.3% 6 months after training. There was no difference in patched eyes of the two groups who gained GA improvement (Table S1).

### Contrast sensitivity (CS)

**Figure 4:**
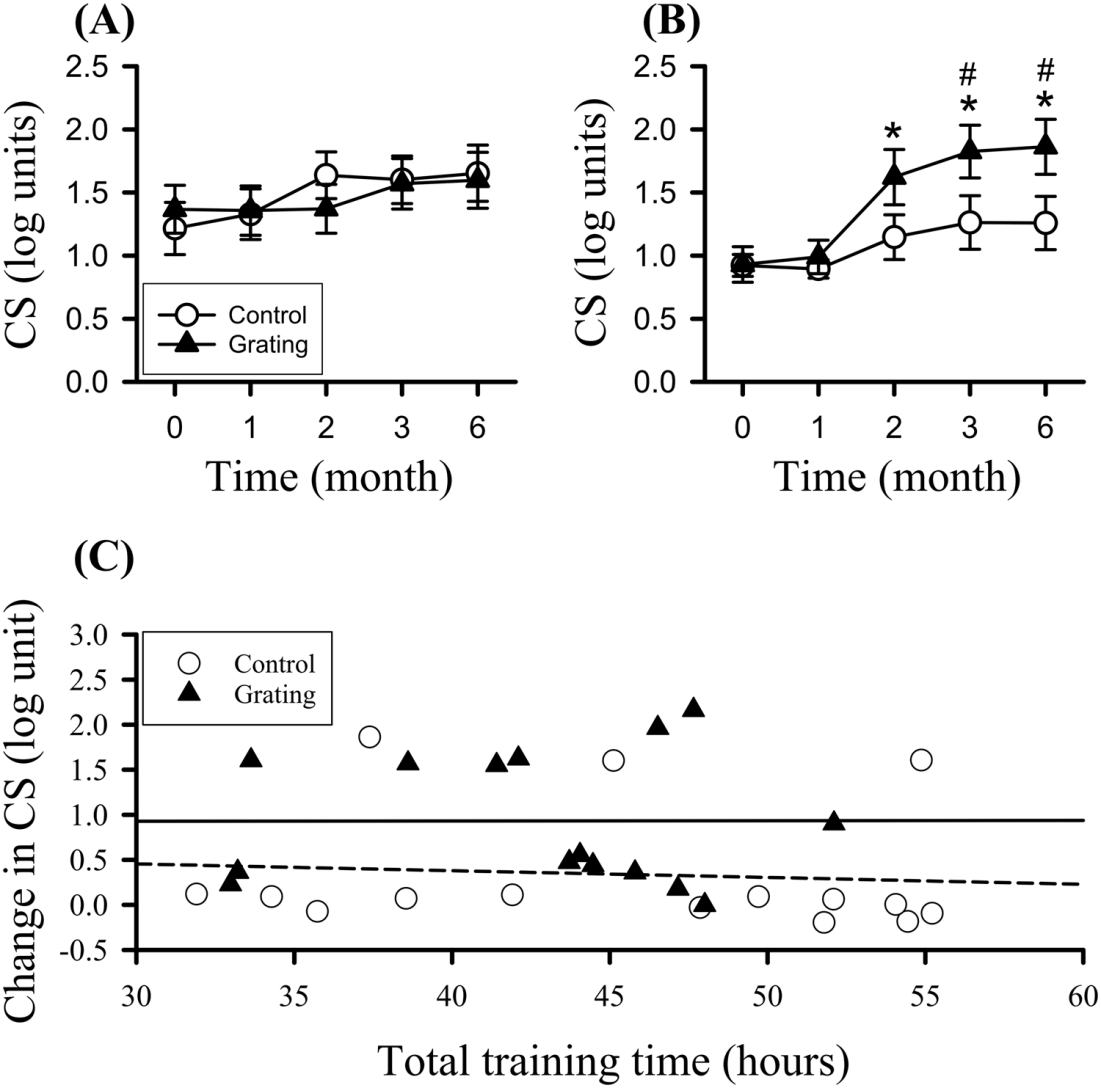
Contrast sensitivity (CS) of 16 cycles per degree (cpd) the baseline (0), 1st, 2nd, 3rd, and 6th months after the training. (**A**) CSs in patched eyes of the control and Grating groups. (**B**) CSs in trained eyes of the control and Grating groups. *p < 0.05 versus baseline, ^*#*^p < 0.05 versus the control group. (**C**) Relationship between 16-cpd CS gain from the 6th month to baseline and total training time in trained eye of the two groups. The long dashed (control) and solid (Grating) lines represent the linear regression line fit the data.

CS spectra of the two groups exhibited a lowpass filter response (Fig. S1 in supplementary results). Frequency response of patched eyes showed no change throughout the training in the two groups. In contrast, trained eyes of the two groups exhibited increased response of 8 and/or 16 cpd. For instance, CS of 8 cpd exhibited significant increase throughout training in the two groups (supplementary results). CS of 16 cpd showed no difference in patched eyes of the two groups (Fig. 4). Most specifically, CS of 16 cpd in trained eyes of the Grating group exhibited significant difference in the factors of time (F(4,112) = 13.93, p < 0.001) and group × time (F(4,112) = 2.69, p = 0.03), but CS of 16 cpd in trained eye of the control group showed no difference. CS of 16 cpd in trained eye of the Grating group was significantly higher at the 2nd, 3rd and 6th months compared with its baseline. Trained eye of the Grating group had increased contrast threshold of 0.93 ± 0.08 log units from baseline to the 6th month. Moreover, trained-eye CS of the Grating group at the 3rd and 6th months was significantly higher than that in the control group. Furthermore, we found CS improvement, particularly for the 16 cpd, took place at the amplyopic eye of the Grating group compared with that of the patched eye throughout the CAM training (Figure S2 in supplementary results).

Relationship between 16-cpd CS gain from the 6th month to baseline and total training time in trained eye of the two groups was analyzed (Fig. 4C). In trained eye of the control group, there was no significant correlation between 16-cpd CS gain and total duration (r = -0.08). In trained eye of the Grating group, there was little correlation (r = 0).

The two groups had no severe deterioration of CS throughout the training. Participants who gained CS improvement of ≥ 0.3 log units in trained eyes of the two groups were counted (Table 2). Increased CS only occurred in 20% of the control group. In contrast, participants with better CS improvement exhibited a progressive elevation to 80% 6 months after training. Participants with gained contrast threshold at the 3rd (χ^2^(1) = 4.89, p = 0.02) and 6th (χ^2^(1) = 8.53, p = 0.003) months were significantly different in the two groups. There was no difference in patched eyes of the two groups who gained CS improvement (Table S1).

## Discussion

All recruited anisometropic amblyopic children completed 6-month training using a portable tablet. Patched eyes of both groups exhibited little change on CS of 16 cpd or progressive improvement in BCVA and GA during training. In a sharp contrast, trained eyes of both groups had significantly progressive improvement in all indexes. In particular, the Grating group exhibited significantly better BCVA, GA, and CS compared with those of the control group at the 6th months of training. Moreover, proportion of the Grating group with improved BCVA (≤ -0.3 logMAR) or CS (≥ 0.3 log units) was significantly higher better than that of the control group at the end of the training. Our results indicate that a convenient portable CAM device with a challenge game flow benefits on great improvement of visual function in anisometropic amblyopic children.

Current available therapies for amblyopia usually take several months to years. A long-time training often faces a failure or dropout issue. Full-time occlusion of the better eye for 3–18 months achieves stable and significant improvement of visual function^9,28^. However, full-time occlusion therapy is a tedious and educationally disruptive technique, and its dropout rate is high (45%-55%)^29,30^. A CAM machine is built for a part-time occlusion. Previous studies have indicated that a CAM training for 2–6 months exhibits significant improvement of visual function^9,20,31^. Because the CAM machine is primarily available in a hospital, patients need to visit hospital frequently. Consequently, its dropout rate is high (56%)^9^. Even though a traditional CAM machine can set up at home, training failure often occurs because the CAM machine contains stereotyped training materials lacking of motivation or interest for participants^9,12^. In a video game treatment with portable device, previous studies have indicated a slow or small effect for amblyopia^25^. These studies have reported a considerable dropout rate^32^. The present study provided valuable information of no dropout in a 6-month training period through our designed flow with a challenge game for children in a portable tablet, which contained 1440 diversity pictures, valuable drawing performance per day, and all assessed results throughout the training period. The valuable information about daily performance and assessments elicited a positive driving force for participants and parents. Interestingly, trained eye of the Grating group exhibited obvious progress (an improvement of ≤ -0.3 logMAR in BCVA) and 80% of the Grating group attained BCVA of ≤ 0.1 logMAR throughout a 6-month training. From tablet record of daily drawing improvement and increased visual function, physician could explain the exciting increased performance to amblyopic children and their parents, which create driving force of self-realization values for patients to continuous playing our game in home. These exciting findings are beneficial to boost participant’s motivation using the CAM-like tablet and to keep low dropout.

Numerous factors affect training performance. Firstly, spatial frequency and/or rotating ability of the grating stimulation in a CAM device is diverse. A fixed-grating stimulation with a single spatial frequency (0.3 cycle/degree) with no rotation only exhibits slight improvement of VA (~ 0.1 logMAR) for a 6-month training^21^. A square-wave rotating grating stimulation with spatial frequencies of 0.5–32 cycle/degree induces improved VA of ~ 0.3 logMAR after 1-month training^9^. Video game studies with absence or a single frequency of grating show a slight VA improvement (0.13–0.21 logMAR)^25^. The current study showed a significant improvement in BCVA (≤ -0.3 logMAR) after training via rotating grating stimulation with 5 spatial frequencies. In particular, the Grating group exhibited significant increase in CS of 16 cpd, which may contribute to increase VA. Intuitively, functional enhancement of VA seems to be related to contribution of rotating property and various spatial frequencies in grating stimulation of a CAM device. Rotating the grating stimulus is able to activate a large range of orientation-specific neurons in the visual cortex^33,34^, which plays an important role in visual processing. Grating stimulation with various spatial frequencies is beneficial to drive a wide-band visual flow of the cortex. Taken together with these two advantages, the present study provides additional evidence to support CAM’s advantages on amblyopia.

Secondly, age of the amblyopic participant influences training performance. In general, child's vision continuously develops until 2 to 3 years old^35^ then slows down until its complete maturation at 8 years old^36^. The previous study has reported that 92% of amblyopic children at 6–9 years old exhibits improved VA of ~ 0.3 logMAR using full-time occlusion therapy, but only 33% of > 9 years old amblyopic children attains comparable VA enhancement^37^. Averaged 6-month grating training with part-time occlusion therapy has little effect in VA and CS for amblyopic patients of 8–17 years old^22^. The present study recruited amblyopic children of 4–8 years old and found 53.3–80% of children with significant improvement in all assessments 6 months after grating training (Table 2). Our results indicate a golden period for amblyopic treatment. Based on these findings, early diagnosis and treatment will be crucial for amblyopia.

Thirdly, characteristic of amblyopia participants perhaps determines effect of CAM treatment. A study exhibits improvement VA (~ 0.1 logMAR) for strabismic or mixed amblyopia^1^. CAM treatment has little effect on deprivation amblyopia^38^, which may arise from unclear images falling on the retina of one or both eyes due to ptosis or congenital cataract^2^. Anisometropic amblyopia has better VA enhancement (~ 0.3 logMAR) compared with strabismic amblyopia (~ 0.1 logMAR) for CAM treatment^31^. Abnormal anatomical change of the strabismic or deprivation amblyopia, e.g., extraocular muscle imbalance, causes ocular misalignment or obstruction in the visual axis, and these structure alterations absolutely reduce the effectiveness of sensory-related CAM treatment. The present study recruited anisometropic amblyopia exclusively to reduce variability from amblyopia attributes. Interestingly, all indexes (i.e., BCVA, GA, and CS) in the Grating group exhibited significant improvement throughout CAM stimulation and improved BCVA of ≤ -0.3 logMAR occurred at 53.3% of children (Table 2). The present study provides supporting evidence on a CAM-like training being beneficial for anisometropic amblyopic children.

Numerous factors result in success of a CAM stimulator on amblyopia, such as increased learning motivation of portable CAM device, wide frequency ranges of rotating grating stimuli, and selection of young children with anisometropic amblyopia exclusively, in previous paragraphs. Moreover, our results have indicated that CAM stimuli produced a slow progression in all outcomes within 6 months. Previous studies used CAM training of < 10 weeks have no significant effect in amblyopia^13,19,39^. These results suggest a long-term CAM training being important in successful intervention for amblyopia. Besides, experimental design with a better sham group is also beneficial to simplify the question and increase effect size. The present study designed two groups with the presence or absence of grating stimulation under drawing, which is a clear setting to test successfulness of CAM stimulation. Previous studies with incomparable or inadequate control group (such as watching television^17^, full-time or part-time occlusion therapy^18^) or no control group^9,16^ exhibit no significant effect. Taken together, the current study provides insight to demonstrate successfulness of a CAM stimulation on amblyopia.

A number of previous studies have indicated poor CS for amblyopia after treatment even patients with a nearly normal acuity^40,41^. A previous study using therapy of occlusion and acuity exercises has indicated that CS of the amblyopic eye is poor even though amblyopic patients express a normal BCVA on both eyes^40^. In contrast, previous studies dealing with perceptual learning have indicated significant improvement in VA and CS^42,43^. Our results showed that amblyopic eye exhibited a poor CS (particularly for 8 and 16 cpd) at the baseline compared with a better-seeing eye. We also observed a progressive improvement in the 3 visual assessments throughout the training. In particular, amblyopic eye showed significantly higher CS of 16 cpd stimulation after 6 months CAM treatment than that of the better-seeing eye (Figure S2). Previous studies used sinusoidal grating stimulation have indicated remarkable improvement of visual acuity and CS after intensive practices^43^. These results have indicated that grating stimulation has positive effect on visual functional improvement.

Patched eye of the Grating group exhibited significant improvement on BCVA and GA rather than CS at the latter stage of CAM treatment. BCVA and GA emphasize on spatial characteristics of stimulated materials, and CS deals with intensity variation of particular spatial frequencies. The discrepancy between BCVA/GA and CS effects of CAM stimulation may arise from different emphases of these measures. A previous study has shown that non-trained eye exhibits slight VA improvement in amblyopic patients throughout a 2-month training^31^. Obviously, patched eye receives little CAM stimulation during treatment. Inter-ocular transfer of the perceptual learning may be a reason to explain the phenomenon of non-training induced enhancement^43^. Another possibility may arise from increased levels of visual attention throughout the training^34^.

Trained eye of the control group here exhibited significant improvement on BCVA, GA and CS of 8 cpd, particularly at the latter stage of the training. This progressive improvement of BCVA is comparable with near-distance outcome of a large amblyopia population from the Pediatric Eye Disease Investigator Group^17^. The phenomenon of improved visual function in the control group may arise from progressive development of visual function throughout a 6-month treatment. Participants fully adapted to their glasses may be a factor to produce the latter progression of BCVA. Practice effect may also contribute to progressive elevation in 5 repetitive measures of the control group. The other possibility may be due to use huge and diverse drawing practice of 1440 pictures. A small set of fairy tales pictures stimulation results in a limited visual improvement^12^. In contrast, a television game overlapping with varying size, orientation, and movement of stripes for 8–12 weeks produces improved VA of 0.1–0.3 logMAR^20^. The results imply stripe stimulation of different sizes, shapes, and orientations, which probably activates a large range of orientation-specific neurons in the visual cortex during drawing eye-hand practice in the control group.

The control group had no correlation between gain of BCVA, GA, or CS and total training time. The results may indicate a placebo effect in the control group. The Grating group showed no correlation between gain of GA or CS and total training time. The Grating group showed a higher BCVA gain as total training time increased. The results suggest a dose–response learning in BCVA, which be associated with a dose–response curve between VA change and occlusion treatment duration^44^. The dose–response relation between BCVA gain and total training time needs more samples to strengthen the correlation.

All children wore glasses with a BCVA prior to the training. BCVA of the Grating group seemed to be slightly worse than the control group initially but there was no difference (Figs. 2). Finally, the Grating group exhibited significant improvement in the 3 visual assessments compared with the control group throughout 6-month training. However, the current study had no record on pre-training duration of optical correction to rule out the possible confounding effect of glasses. A previous study suggests ≥ 16 weeks of spectacle wear prior to enrollment to minimize the impact of improvement with glasses alone^45^. It needs to raise attention on the issue of pre-training glasses wearing in the future.

The present study demonstrated significant visual enhancement for anisometropic amblyopic children using a tablet-based CAM training system in a small sample size (n = 30). More participants will be needed to generalize our results using a tablet with rotating grating stimuli. Moreover, full-time occlusion therapy is widely used in amblyopia treatment and produces a valuable contribution^31^. Probably, the tablet-based CAM treatment combined with full-time occlusion therapy or other therapies^10^ may facilitate visual enhancement particularly at an early stage of treatment.

## Materials and methods

### Participants

Thirty-five participants were recruited from the National Cheng Kung University Hospital and Chiayi Chang Gung Memorial Hospital. The study is registered at ClinicalTrials.gov (NCT04213066, 30/12/2019). The experimental procedure was reviewed and approved by the Institutional Review Boards of the National Cheng Kung University Hospital and Chiayi Chang Gung Memorial Hospital institutes. Informed consent was provided and signed for all amblyopic participants and their parents before the experiment. All methods were performed in accordance with the relevant guidelines and regulations.

Three inclusion criteria were as follows: 1.) Participants were 4–8 years old and were diagnosed anisometropic amblyopia. 2.) Participant had binocular or monocular best-corrected VA (BCVA) of ≥ 0.1 logMAR, or they exhibited binocular BCVA difference of ≥ 0.2 logMAR^1^. 3.) Participant had the wearing of optimal spectacle correction. Participants who displayed deprivation amblyopia, manifested strabismus, or had ever eye surgery were excluded. In the present study, five participants with deprivation amblyopia were excluded (Fig. 1). Thirty anisometropic amblyopic participants were randomly assigned into the control group without grating stimulation or the experimental group with rotating grating stimulation (Grating). Participants and testers who measured outcomes were masked to treatment allocation.

### Experimental procedure

**Figure 5:**
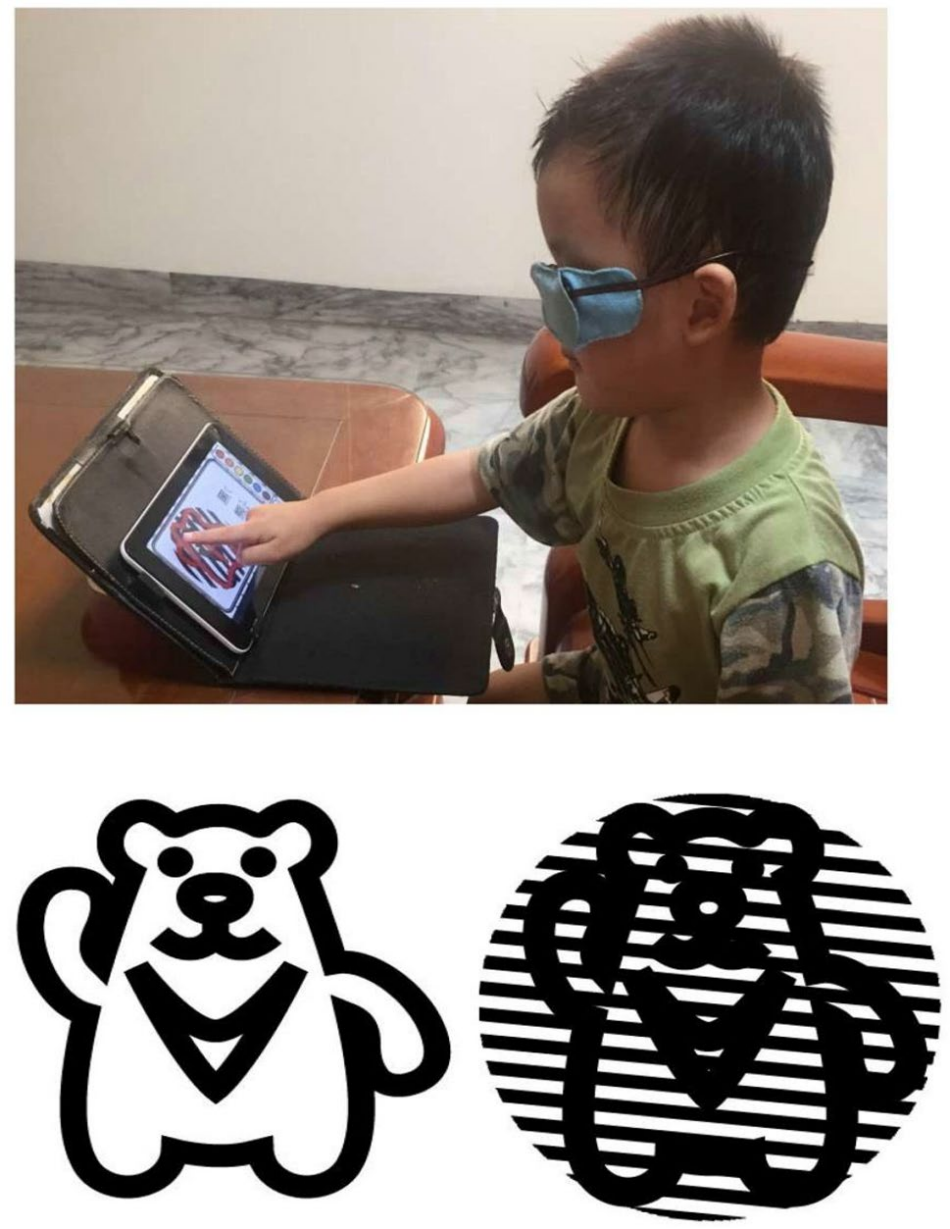
Example of an amblyopic child wearing a patch and playing the training tablet. Bottom panel illustrates two stimuli for the control group (left) and the Grating group (right) respectively.

The experimental procedure contained two primary parts: training and evaluation. Occlusion with an eye patch over a better-seeing eye of the two groups during training, which forced them using weaker or amblyopic eye (trained), was used to potentiate the practice effect for amblyopic eye^28^. An eye patch attached to the glasses was used to avoid skin irritation and was used for anti-peep design. All participants drew the contour of an object in our developed training system on a tablet. The control group saw picture and drew, and the Grating group drew pictures superimposed with rotating grating stimulation of various spatial frequencies (Fig. 5). The parent of the participant provided a signed consent to publish form for Fig. 5. Participants trained ≥ 1 session a day for 5 days a week. To avoid overuse of a trained eye of each individual, each training time was 15 min a session. The entire training period was 6 months. Participants were scheduled for visual evaluation (BCVA, GA, and CS) and downloading training records, i.e., training frequency and duration from their tablets at the baseline, 1st, 2nd, 3rd, and 6th months (Fig. 1).

### Training system

A home-based training system was implemented on a general tablet (MD7072, Ergotech Inc., Taipei, Taiwan), which was equipped with a 7-inch display (resolution: 800 × 480 pixels) and operated under the Android 4.0.4 platform. The training element was based on a CAM-like concept^9^. Participants seated an arm distance (35–40 cm) from the tablet. Grating stimuli with various frequencies (1, 2, 4, 8 and 16 cycle/cm) were rotating as previous studies did^9,11^. The training system contained 1440 pictures and categorized into 12 sets in terms of traditional Lunar Chinese Animals. Each set contained 6 clusters, and each cluster consisted of 20 pictures with similar spatial frequencies. Proportion of higher spatial frequency of the grating stimulus was progressively decreased as the set number increased. When participants exhibited consistency of > 70% between drawing and pictures, the training system allowed users to choose a new picture, cluster, or set. Consistency between drawing and picture accompanied by previous records were shown at the end of a daily training. Participants and their parents could explicitly know progress of the eye-hand coordination to promote their learning motivation. Moreover, this system also recorded total training time and total training session as an objective monitoring of amblyopic training. When participant revisited the hospital, physician was able to read out their performance each month and provided constructive feedbacks.

### Visual evaluation

Each participant’s best refractive correction was determined by cycloplegic subjective refraction, and the appropriate full correction was ascertained for all subsequent tests. All test procedures were conducted in the same clinic facilities under identical lighting conditions.

BCVA was measured for amblyopia and was using an 8-AFC Landolt C (LC) chart along rows of five LCs, which size decreased from top to bottom. A forced-choice procedure was used under monocular viewing condition. A participant sat 5 m away from the stimulation and indicated the orientation of the gap, either verbally or by pointing in that direction. The children could take as long as they wanted to respond. Acuity of visual stimulus was presented by an up-staircase procedure of 0.1. A LC row containing 5 letters was selected and displayed. Participants were asked to answer 5 LC letters from left to right, and three correct orientations of C rings were required for its reliability. The test was aborted if three incorrected answers occurred, then participant’s BCVA was obtained. BCVA score was in logMAR units. A lower LC score means a better BCVA^46^. The reliability of BCVA measure has been verified^47^.

The stimuli of GA and CS were generated by a laptop (Microsoft visual studio program) and presented on an 11-inch LCD monitor (1280 × 800 pixel). We measured GA with circular shape of horizontal or vertical square-wave grating (600 × 600 pixel) for amblyopia^48^. Spatial frequency ranged from 13 to 38 cycle/degree (cpd) in regard to alter distance of a laptop from participant as 1 to 3 m. Participant was asked to report the orientation of the grating (i.e., horizontal or vertical), which presented randomly. Acuity of grating stimulus was presented by staircase procedure of 1 cpd^22^. Each acuity presented 5 times for reliability and recorded participants’ answers. Acuity level was increased when a participant could correctly report the grating orientation ≥ 4 of 5 trials as threshold. The children could take as long as they wanted to respond. GA score was considered as the maximal spatial frequency when a participant attained the threshold^4^. The reliability and validity of GA measure has been verified^49^.

CS was measured for amblyopia by a circular-shape horizontal square-wave grating (600 × 600 pixel) with spatial frequency of 1, 2, 4, 8 and16 cpd. Each spatial frequency respectively performed 1 trial for up and down staircase. The contrast of square-wave grating of up and down staircase procedures on an 11-inch LCD was performed. Each contrast lasted for 0.5 s. The method of limits was used to identify contrast threshold. Participants were asked to ascertain the presence of a horizontal grating in the upward manner of stimulus contrast. Inter-trial interval was 10 s. During testing, a participant sat 1 m away from the stimulation. In the present study, Michelson contrast for a pattern, the difference between maximum and minimum luminance divided by the sum of the maximum and minimum luminance ((L_max_-L_min_)/(L_max_ + L_min_))^50,51^, was calculated. Grating CS score was calculated as log unit. Higher log CS score means a better contrast acuity. According to previous results^52,53^, the grating CS of 16 cpd exhibited a better sensitivity for anisometropic amblyopia discrimination. The reliability of CS measure has been verified^54^.

### Statistical analysis

Normality and equal variance tests were assessed before using parametric statistic for all measures. Student t test was used to assess the control and Grating groups with regard to continuous variables including age, BCVA, GA, and CS. Total training time and total training sessions of the groups were compared by Student t test. BCVA, GA, and CS in patched and trained eyes of the control and Grating groups at the baseline (0), 1st, 2nd, 3rd, 6th months were assessed by two-way mixed model analysis of variance, if appropriate, followed by post hoc comparison with Bonferroni correction. Moreover, the number of participants who gained ≤ -0.3 logMAR of BCVA^55,56^, ≥ 10 cpd of GA^4,22^, and ≥ 0.3 log units of CS^57,58^ compared with their baseline respectively was calculated and compared by χ^2^ test in two groups. All statistical analyses were using SPSS version 16.0 software. A two-tailed significance level was set at p ≤ 0.05.

## Supplementary Information

Supplementary Information.
