# Supplementary material for: Portable rotating grating stimulation for anisometropic amblyopia with 6 months training
Source: Sci Rep. 2021 Jun 1;11:11430. doi: 10.1038/s41598-021-90936-7 (PMC8169940; doi:10.1038/s41598-021-90936-7)
Supplement: Supplementary file 1 — Supplementary Information. [file 41598_2021_90936_MOESM1_ESM.docx]

**Portable rotating grating stimulation for anisometropic amblyopia with 6 months training**

**Wen-Hsiu Yeh^1^, Li-Ju Lai^2^, Da-Wei Chang^3^, Wei-Sin Lin^3^, Guan-Ming Lin^3^ & Fu-Zen Shaw^1,4*^**

^1^Institute of Basic Medical Sciences, National Cheng Kung University, Tainan 701, Taiwan

^2^Department of Ophthalmology Chang Gang Memorial Hospital, Chia-Yi Branch Chiayi, 613, Taiwan

^3^Department of Computer Science and Information Engineering, National Cheng Kung University, Tainan 701, Taiwan

^4^Department of Psychology, National Cheng Kung University, Tainan 701, Taiwan

*Corresponding author

Fu-Zen Shaw, PhD

Department of Psychology

National Cheng Kung University

No 1 University Road, Tainan 70101, Taiwan

Tel: +886-6-2757575 ext 56507

Fax: +886-6-2752029

E-mail: [fzshaw@gmail.com](mailto:fzshaw@gmail.com)

**Highlights**

1.) Portable CAM-like device is acceptable with no dropout throughout 6-month training.

2.) The Grating group exhibited significantly higher performance in 3 visual assessments under a randomized controlled trial.

3.) Percentage of the Grating group with a great visual improvement was significantly larger than the control group.

**Supplementary results**

Fig. S1 shows spectra of contrast sensitivity (CS) for patched and trained eyes of the two groups throughout the 6-month training. CSs of both groups exhibited a low-pass filter response regarding to various spatial frequencies. In the control group (Fig. S1A), CS of patched eye showed significant difference in the factor of spatial frequency (F(4,224)=65.98, p<0.001) exclusively. CS of trained eye showed significant difference in the factors of time (F(4,224)=3.53, p=0.012), spatial frequency (F(4,224)=89.17, p<0.001), and their interaction (F(16,224)=1.72, p=0.04). CS of 16 cpd in patched eye of the control group exhibited significantly higher at the 2nd and 6th months compared with its baseline. CS of 8 cpd in trained eye of the control group exhibited significantly higher at the 3rd and 6th months compared with its baseline.

In the Grating group (Fig. S1B), CS of patched eye showed significant difference in the factor of spatial frequency (F(4,224)=55.85, p<0.001) exclusively. CS of trained eye showed significant difference in the factors of time (F(4,223)=3.74, p=0.009), spatial frequency (F(4,223)=52.63, p<0.001), and their interaction (F(16,223)=4.46, p<0.001). CS of 8 cpd in trained eye of the Grating group exhibited significantly higher at the 2nd and 3rd months and almost attained significant difference at the 6th month (t=2.374, p=0.073) compared with its baseline. Moreover, CS of 16 cpd in trained eye of the Grating group exhibited significantly higher at the 2nd, 3rd, and 6th months compared with its baseline.


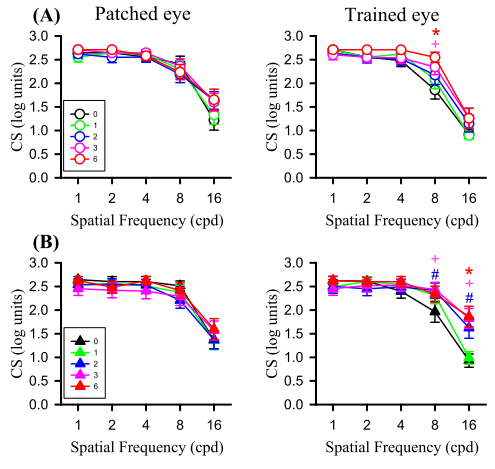


**Figure S1.** CS spectra of the two groups regarding to 5 spatial frequencies throughout the training (the baseline (0), 1st, 2nd, 3rd, and 6th months). (A) CS spectra of patched (left) and trained (right) eyes of the control group. (B) CS spectra of patched (left) and trained (right) eyes of the Grating group. ^#^p<0.05 2nd month compared with baseline, ^+^p<0.05 3rd month compared with baseline, *p<0.05 6th month compared with baseline.

Fig. S2 shows spectra of contrast sensitivity (CS) for patched and trained eyes of the Grating group throughout the 6-month training. CSs of patched and trained eyes at baseline showed significant difference in the factors of spectrum (F(4,56)=53.89, p<0.001), treatment (F(1,14)=8.97, p=0.01), and their interaction (F(4,56)=2.85, p=0.03). CSs of 8 and 16 cpds in patched eye at baseline exhibited significantly higher than those of trained eye. CSs of patched and trained eyes at the 1st month showed significant difference in the factor of spectrum (F(4,56)=41.27, p<0.001). CS of 16 cpd in patched eye at the 1st month exhibited significantly higher than that of trained eye. CSs of patched and trained eyes at the 2nd month showed significant difference in the factor of spectrum (F(4,56)=27.64, p<0.001). CSs of patched and trained eyes at the 3rd month showed significant difference in the factor of spectrum (F(4,56)=14.66, p<0.001). CSs of patched and trained eyes at the 6th month showed significant difference in the factor of spectrum (F(4,56)=16.78, p<0.001). CS of 16 cpd in patched eye at the 6th month exhibited significantly lower than that of trained eye.


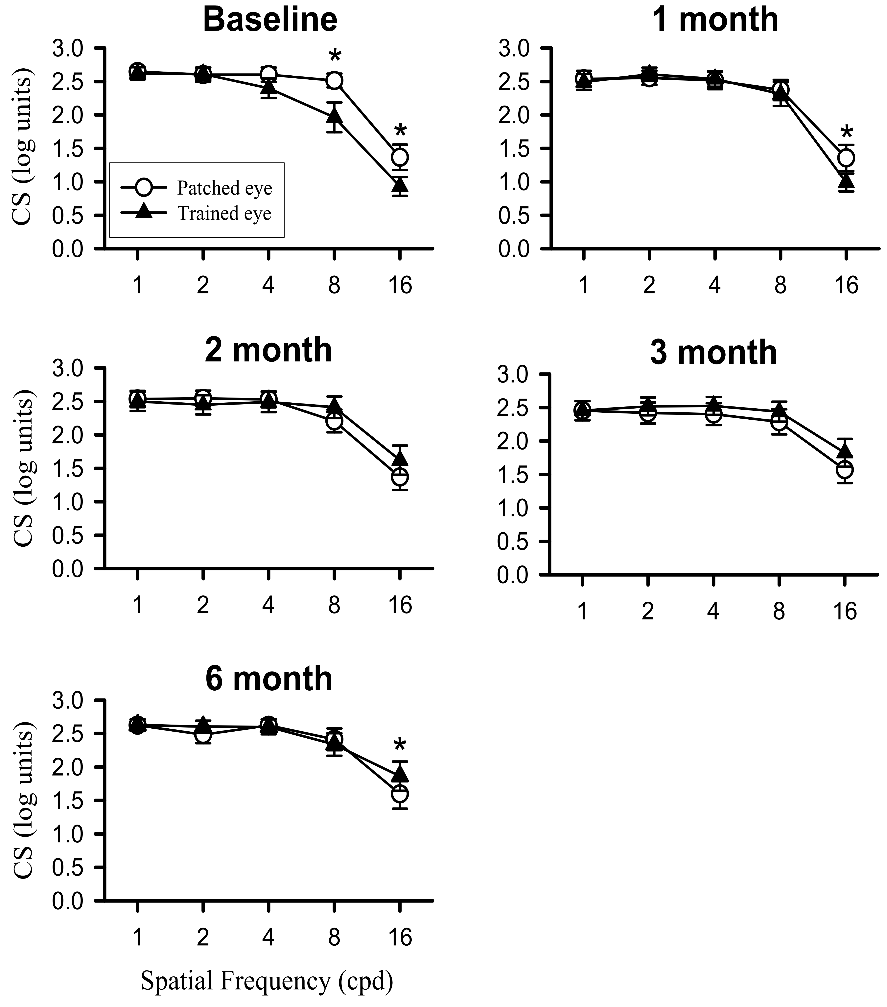


**Figure S2.** CS spectra of the two eyes of the Grating group regarding to 5 spatial frequencies at the baseline (0), 1st, 2nd, 3rd, and 6th months. *p<0.05 patched eye compared with trained eye.

Table S1 shows gained BCVA, GA, and CS of patched eye at the 1st, 2nd, 3rd, and 6th months after the training in the two groups. Participants who gained BCVA of ≤-0.3 in patched eyes of the two groups were counted. Improved BCVA occurred in at most 6.7% of the control group. Improved BCVA exhibited in 13.3% of the Grating group 6 months after training.

Patched eyes of the two groups showed no severe deterioration of GA throughout the training. Increased GA occurred in 26.7% of the control group. Participants with increased GA exhibited a progressive elevation to 33.3% 6 months after training. In addition, participants who gained CS of ≥0.3 in patched eyes of the two groups were counted. Increased CS occurred in 53.3% of the control group. Participants with increased CS exhibited a progressive elevation to 40% 3-6 months after training.

|  | **Control** | **Grating** |
| --- | --- | --- |
| **BCVA (≤-0.3)** |  |  |
| 1 month | 0/15=0% | 1/15=6.7% |
| 2 month | 1/15=6.7% | 1/15=6.7% |
| 3 month | 1/15=6.7% | 1/15=6.7% |
| 6 month | 1/15=6.7% | 2/15=13.3% |
| **GA (≥10 cpd)** |  |  |
| 1 month | 0/15=0% | 0/15=0% |
| 2 month | 1/15=6.7% | 0/15=0% |
| 3 month | 3/15=20% | 1/15=6.7% |
| 6 month | 4/15=26.7% | 5/15=33.3% |
| **CS (≥0.3)** |  |  |
| 1 month | 3/15=20% | 2/15=13.3% |
| 2 month | 8/15=53.3% | 4/15=26.7% |
| 3 month | 8/15=53.3% | 6/15=40% |
| 6 month | 8/15=53.3% | 6/15=40% |
| **Table S1.** Participants have gained better BCVA, GA, and CS of patched eye at the 1st, 2nd, 3rd, and 6th months after the training in the control and the Grating groups. | | |
